# Characteristics of resting state functional connectivity of motor cortex of high fitness level college students: Experimental evidence from functional near infrared spectroscopy (fNIRS)

**DOI:** 10.1002/brb3.3099

**Published:** 2023-06-12

**Authors:** Wenyi Li, Qi Zhang, Rui Yang, Bin Liu, Gonghe Chen, Bingyang Wang, Taiyu Xu, Jun Chen, Xuxue Zhou, Shilin Wen

**Affiliations:** ^1^ Department of Physical Education and Training Capital University of Physical Education and Sports Beijing China; ^2^ College of Physical Education and Sport Langfang Normal University Langfang Hebei China; ^3^ School of Physical Education Baise University Baise Guangxi China; ^4^ Department of Physical Education Institute of Disaster Prevention Sanhe Hebei China; ^5^ Department of Physical Education Changsha Medical University Changsha Hunan China

**Keywords:** fitness level, fNIRS, motor cortex, resting state functional connectivity

## Abstract

**Objective:**

This study inspects difference of resting state functional connectivity (RSFC) of motor cortex between athletes and ordinary college students and the test‐retest reliability of RSFC.

**Methods:**

Twenty high fitness level college students (high fitness group) and 20 ordinary college students (control group) were recruited. The motor cortical blood oxygen signals in resting states were monitored by functional near infrared spectroscopy (fNIRS). RSFCs of brain signals were preprocessed and calculated by FC‐NIRS software. RSFC results of test‐retest reliability were evaluated by intra‐class correlation coefficient (ICC).

**Results:**

Total RSFC (HbO signal) was significantly different between high fitness group (0.62 ± 0.04) and low fitness group (0.81 ± 0.04) (*p* < .05). Significant differences were found between the groups (HbO signal) in 50 edges among the 190 edges of motor cortex (14 edges after FDR corrected). At three hemoglobin concentrations, mean of group‐level *ICC* (*C*, 1) for total RSFC in two groups was 0.40 ± 0.10, whereas the mean of group‐level *ICC* (*C*, *k*) was 0.57 ± 0.11, depicting “fair” reliability. The mean of group‐level *ICC* (*C*, 1) of 190 “edges” was 0.88 ± 0.06, whereas mean of *ICC* (*C*, *k*) was 0.94 ± 0.03, exhibiting “excellent” reliability.

**Conclusion:**

Fitness level is the factor causing specific changes in RSFC strength of motor cortex that can be utilized as biomarker for evaluating the fitness level.

## Graphical abstract

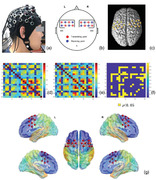

## 1 INTRODUCTION

Fitness level refers to the ability of physical activity of an individual in sports which is the embodiment of an individual's physical development. It can be evaluated by the level of sports skills, training years, and participation in competitions (Mckay et al., 2022). Mckay et al. (2022) divided fitness level into 6 levels according to exercise background and athletic ability: Level 0: sedentary; Level 1: recreationally active; Level 2: trained/developmental; Level 3: highly trained/national level; Level 4: elite/international level; and Level 5: world class. This classification describes population proportion, activity time, competition specifications, skills, and other aspects of individuals from each level. This in turn provides a scientific theoretical framework for evaluating the people of various fitness levels.

The exercise level is related to individual's innate genetic factors and also influenced by the physical activity (Perusse et al., 1989). Long term regular and specialized physical or skill training positively affects body functions, including the increased muscle strength, bone density, cardiopulmonary fitness, nervous system regulation, and so on, thereby enhancing the individual exercise performance. Higher the exercise levels in addition to outstanding motor performance, better are the cognitive functions, such as working memory (Padilla et al., 2014), inhibitory control (de Almeida‐Net et al., 2022), and attention (Llorens et al., 2015). There is thus a degree of correlation between exercise levels and cognitive ability (Erickson et al., 2019).

Advancements in neuroimaging techniques have assisted in exploring the differences of cognitive function and brain structure between people of diverse fitness levels. Studies have revealed that long term specialized trainings improve the individual fitness level and cause adaptive changes in brain structure and function (Faull et al., 2018; Park et al., 2009; Vints et al., 2022). Regarding the brain structure, high level endurance athletes have larger volumes of white and gray matter in medial temporal lobe (Chang et al., 2015; Schlaffke et al., 2013). Paruk et al. (2020) used functional magnetic resonance imaging (fMRI) for measuring the whole brain and local brain volumes in high level endurance athletes and sedentary individuals. The whole brain analysis exhibited greater gray and white matter volumes and total brain volume in high level athletes, whereas local analysis revealed smaller gray matter volumes in areas, such as right primary sensory and motor cortex, the middle inferior frontal gyrus, and the left thalamus. The fMRI study for brain function depicted that college football players had greater activation in oculomotor and prefrontal oculomotor regions of cerebellum during a visual smooth pursuit task (Kellar et al., 2018). There may be a dynamic bilateral relation between behavior changes and intrinsic physiological changes in the brain (Loprinzi et al., 2013). Brain has a role in regulating the motor behavior and exercise performance, while exercise training, in turn, affects brain function and structure. The brain regulates motor behavior and performance while motor training influences brain function and structure. Early changes observed in the brain because of exercise are mainly the cerebral blood flow and metabolic activity, and later there are structural changes in local brain regions (Casanova, 1997). Changes in brain function may occur earlier than the macroscopic changes in brain structure. It is thus imperative to explore the changes in internal brain function of athletes for understanding their external behavior and promoting behavioral performance through intrinsic neural regulation.

In recent years, resting state functional connectivity (RSFC) based on neuroimaging algorithms is employed as a biomarker for evaluating the low‐frequency fluctuations in brain and subsequently indicate the changes in brain function and provide evidence for “exercise‐brain” effect (Zhang et al., 2012). Li et al. (2014) demonstrated that RSFC between medial prefrontal cortex and medial temporal lobe was changed in the experimental group compared to the control, and RSFC strength between these regions was correlated to individual cognition. RSFC is adopted in human brain connectomics because of its principles and computational simplicity in exploring the relation between movement and cognition.

Motor cortex is the main functional brain area for planning, control, and execution during the exercise (Postle, 2015). Can motor cortex RSFC be employed as a valid biomarker for evaluating the level of movement? The functional near infrared spectroscopy (fNIRS) is utilized to investigate the characteristics of motor cortical RSFC in athletes (training/developmental level) and general college students (sedentary). This study hypothesizes that fitness level causes specific changes of RSFC in motor cortex that can act as biomarker in evaluating the fitness level.

## 2 MATERIALS AND METHODS

### 2.1 Participants

Twenty track and field athletes from a sports university in Beijing were recruited to high fitness group (H Group), and 20 ordinary college students to low fitness group (L Group). The basic information of subjects is given in Table 1. Experimental assistant explained the study protocol to proposed participants by telephone or e‐mail, and consented individuals were included to the study. Inclusion criteria: normal intelligence, no history of brain injury or psychiatric disorders; no flu symptoms, no major mood swings; right‐handedness; no alcohol consumption, asked to be well‐rested, and no heavy exercise or physical work in 3 days prior to experiment; participants in H Group held a certificate of National Sports Skill Level‐2 or above and were preparing for the provincial or national collegiate competition, which met the “trained/developmental level” criteria of Mckay et al. (2022); subjects in L Group had not reached the minimum physical activity level and were occasionally physically active, satisfying the criteria of “sedentary level” (Mckay et al., 2022). From initially recruited 46 subjects, 42 were left after the inclusion screening, including 22 in H Group (4 were excluded) and 20 in L Group.

**TABLE 1:** Demographic data.

Variables	H Group (*n* = 20)	L Group (*n* = 20)	*df*	*t*‐Value	*U*‐Value	*Z*‐Value	*p*‐Value
Gender	Male	Male	–	–	–	–	–
Age (years)	20.00 (19.25,20.00)	20.00 (19.25,22.75)	–	–	145.00	−1.59	.14
Education (years)	14.00 (14.00,14.00)	14.50 (14.00,15.75)	–	–	139.50	−1.93	.10
Height (cm)	180.00 ± 6.76	176.60 ± 5.20	38	1.8	–	–	.08
Weight (kg)	70.85 ± 7.65	71.60 ± 8.08	38	−0.30	–	–	.77
BMI (kg/m^2^)	21.83 ± 1.71	22.92 ± 1.92	38	−1.89	–	–	.07

*Note*: Normally distributed measurement data are described as mean (±SD) and non‐normally distributed are described as Me (Q1 and Q3).

Abbreviations: BMI, body mass index; H Group, high fitness group; L Group, low fitness group; Me, median; Mean, arithmetic mean; Q1, the first quartile; Q3, the third quartile; SD, standard deviation.

Twenty‐two subjects participated in H Group for the experiments and two more were excluded because of the low signal‐to‐noise ratios (SNRs) in some of measured channels (less than 25% of average SNR of all channels), resulting in the data of 20 samples. Participants signed informed consent before the experiments. The study received ethical approval from Ethics Committee of Capital University of Physical Education and Sport (ID 2020A07). The methods were executed as per the latest guidelines and regulations of Declaration of Helsinki.

Mann–Whitney *U*‐test depicted no significant differences in age (*U* = 145.0, *Z* = −1.59, *p* = .142) and years of education (*U* = 139.5, *Z* = −1.93, H Group: *p* = .102) between the two groups. Independent sample *t*‐tests revealed no significant differences in height (H Group: 180.00 ± 6.76, L Group: 176.60 ± 5.20, *p =* .083), weight (H Group: 70.85 ± 7.65, L Group: 71.60 ± 8.08, *p* = .765), and BMI (H Group: 21.83 ± 1.71, L Group: 22.92 ± 1.92, *p* = .067) for the two groups.

### 2.2 The fNIRS monitoring program

fNIRS image (Nissan Shimadzu Portable Light NIRS) was utilized for acquiring the blood oxygen signals from pas at sampling frequency of 10 Hz, 20 channels, and 3 wavelengths (780, 805, and 830 nm). Subjects completed the 5 min resting state scan of motor cortex during which they were asked to close eyes but not to fall asleep. The procedure was as follows:

#### 2.2.1 The fNIRS operation procedure

The fNIRS photopolar cap was worn. The main recording site was found. Subjects were seated. According to the international 10–20 system, intersection point between the nasion to occipital tuberosity and bilateral external auditory foramen was determined as Cz. No. 7, light emitter and No. 2 light detector were placed 1–2 cm before the Cz point, and fNIRS topographic map measurement panel was covering the motor cortex, see Figure 5a–c. The measurement panel consisted of two 2 × 4 photoprobes (including four photodetectors and four light emitters with probe spacing of 3 cm) generating 20 channels (Channels, CH). Brain areas of 20 channels were distributed as follows: first row (CH1, CH2, CH3, CH11, CH12, and CH13); second row (CH4, CH5, CH6, CH7, CH14, CH15, CH16, and CH17); and third row (CH8, CH9, CH10, CH18, CH19, and CH20). After wearing the caps, probe sets were checked and adjusted to ensure that all participants were wearing at the same position.

Signal strength of the channels was checked. Channel signal was affected by the cleanliness of probe, hair, and scalp oil. Channel signal strength was thus checked by the fNIRS system before monitoring. The following measures were taken if CH signal strength was weak: probe cleaning, and adjusting the position of probe jam and the contact area between probe and scalp. The signal emission strength could be further adjusted via fNIRS system if signal strength after the adjustment was still unsatisfactory.

Brain area localization and MNI spatial alignment. The exact photodetector position on the scalp was measured using 3D localizer. NIRS_SPM was employed to confirm the registration of standard brain template and 3D location, and then MNI coordinates of each channel and corresponding brain region of channel were obtained, see Table 2.

**TABLE 2:** MNI coordinates and corresponding anatomical positions of functional near infrared spectroscopy (fNIRS) channels.

	MNI			
Channels	*X*	*Y*	*Z*	Region
1	−59	−2	43	Premotor and supplementary motor cortex_L
2	−44	6	58	Premotor and supplementary motor cortex_L
3	−27	6	69	Premotor and supplementary motor cortex_L
4	−65	−19	41	Primary sensory‐motor cortex_L
5	−51	−11	57	Primary motor cortex_L
6	−35	−7	67	Premotor and supplementary motor cortex_L
7	−14	−8	77	Premotor and supplementary motor cortex_L
8	−57	−26	55	Primary sensory‐motor cortex_L
9	−42	−20	68	Primary motor cortex_L
10	−24	−18	76	Premotor and supplementary motor cortex_L
11	25	12	69	Premotor and supplementary motor cortex_R
12	45	13	58	Dorsolateral prefrontal cortex_R
13	57	13	39	Shimagabe_R
14	13	−4	76	Premotor and supplementary motor cortex_R
15	34	‐1	66	Premotor and supplementary motor cortex_R
16	52	−2	56	Premotor and supplementary motor cortex_R
17	64	2	37	Premotor and supplementary motor cortex_R
18	22	−15	76	Premotor and supplementary motor cortex_R
19	42	−14	69	Premotor and supplementary motor cortex_R
20	58	−14	54	Primary sensory‐motor cortex_R

*Note*: L is the left and R is the right hemisphere of brain.

#### 2.2.2 The fNIRS data processing

The acquired raw data were imported to light NIRS analysis system. Blood oxygen signals of HbO, HbR and HbT, and light intensity signals were converted to TXT format and saved. Preprocessing of exported raw data and RSFC calculations were made through FC‐NIRS software package. (1) Quality control: The motion artifacts were detected by quality control module, SNR was calculated, the bad leads were calibrated, and unqualified data were eliminated. (2) Preprocessing: The light intensity signals of each channel were converted to HbO, HbR and HbT concentration signals by applying modified Beer–Lambert law (Xu et al., 2015). A band‐pass range between 0.01 and 0.1 Hz was used to eliminate the low‐frequency noise. The spline interpolation and correlation‐based signal improvement were employed to reduce motion‐induced artifacts. A least‐square fit of straight line corrected the linear detrending (Xu et al., 2015). (3) Functional connectivity calculation: The average correlation coefficient (Pearson's *r* value) of two connected channels was calculated to attain RSFC matrix for all channels, and further RSFC values were calculated at the groups level. BrainNet Viewer software imaged the “edges” where there were differences between the groups (*p* < .05) (https://www.nitrc.org/projects/bnv/).

### 2.3 Statistical analysis

Independent samples *t‐*test compared: (1) total motor cortex RSFC (total level); (2) motor cortex local cortex RSFC for all channels (local level); and (3) intergroup differences for single edge (190 edges in total, channel level). Application of MATLAB data platform for the extraction, statistical analysis, and the visualization of relevant data; and (4) intra‐class correlation coefficients (ICC) were utilized for evaluating the test‐retest reliability of RSFC. ICC was two‐way random effects model based on consistency measures for assessing the reliability of single and average measures, that is, ICC (*C*, 1) and ICC (*C*, *k*).

## 3 RESULTS

### 3.1 Intergroup differences in total RSFC in motor cortex

**FIGURE 1:**
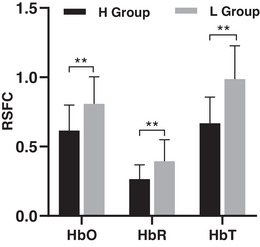
Comparison of total resting state functional connectivity (RSFC). *Note*: ** for *p* < .05.

Independent samples *t*‐test depicted that the difference was significant regarding total RSFC of motor cortex between H Group (0.62 ± 0.18) and L Group (0.81 ± 0.20) at HbO signal level (*p =* .003*, d* = 1.0). Similarly, HbR (H Group: 0.27 ± 0.10, L Group: 0.40 ± 0.16, *p* = .004, *d* = .97) and HbT signals (H Group: 0.67 ± 0.19, L Group: 0.99 ± 0.24, *p* = .000, *d* = 1.49) were significantly different, see Figure 1.

### 3.2 Intergroup differences of RSFC for local areas of motor cortex

**FIGURE 2:**
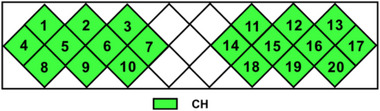
Distribution of channels in each row of motor cortex.

**FIGURE 3:**
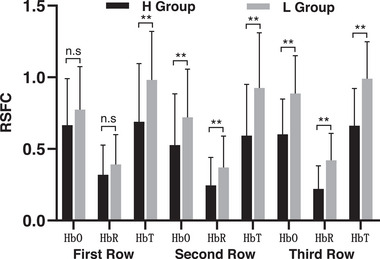
Comparison of resting state functional connectivity (RSFC) in local areas of motor cortex.

RSFC was compared in the local areas of motor cortex (HbO signal), see Figure 2. The intergroup differences for the first row of channels (15 edges in total, CH1, CH2, CH3, CH11, CH12, CH13, measuring brain areas including premotor cortex, supplementary motor cortex, dorsolateral prefrontal cortex, and insula, see Table 2) were not significant (H Group: 0.67 ± 0.33, L Group 0.78 ± 0.30, *p* = .145, FDR corrected, *d* = .35). Intergroup differences in mean RSFC values for the second row of channels (28 edges in total, CH4, CH5, CH6, CH7, CH14, CH15, CH16, CH17, measuring brain areas that may include supplementary motor cortex and premotor cortex) were significant (H Group: 0.53 ± 0.36, L Group: 0.72 ± 0.34, *p* = .005, FDR correction, *d* = .34). Intergroup differences in RSFC for the third row (15 edges in total, CH8, CH9, CH10, CH18, CH19, CH20, measuring brain areas including primary sensorimotor cortex, primary motor cortex, premotor cortex, and supplementary motor cortex, see Table 2) were significant (H Group: 0.61 ± 0.25, L Group: 0.89 ± 0.26, *p* = .001, FDR corrected, *d* = 1.10). Moreover, the intergroup differences were significant in only the first row of HbT signal levels (H Group: 0.69 ± 0.41, L Group: 0.98 ± 0.34, *p* = .000, FDR corrected, *d* = .77). The other rows of HbR and HbT signals were consistent with the HbO signals, see Figure 3. RSFC in motor cortex of college students with high fitness was specific, while excluding the possibility that non‐motor cortical signals could interfere with the findings (e.g., the first row of signal sources may include parts of frontal cortex).

### 3.3 Intergroup differences in single edges of motor cortex (190 edges)

**FIGURE 4:**
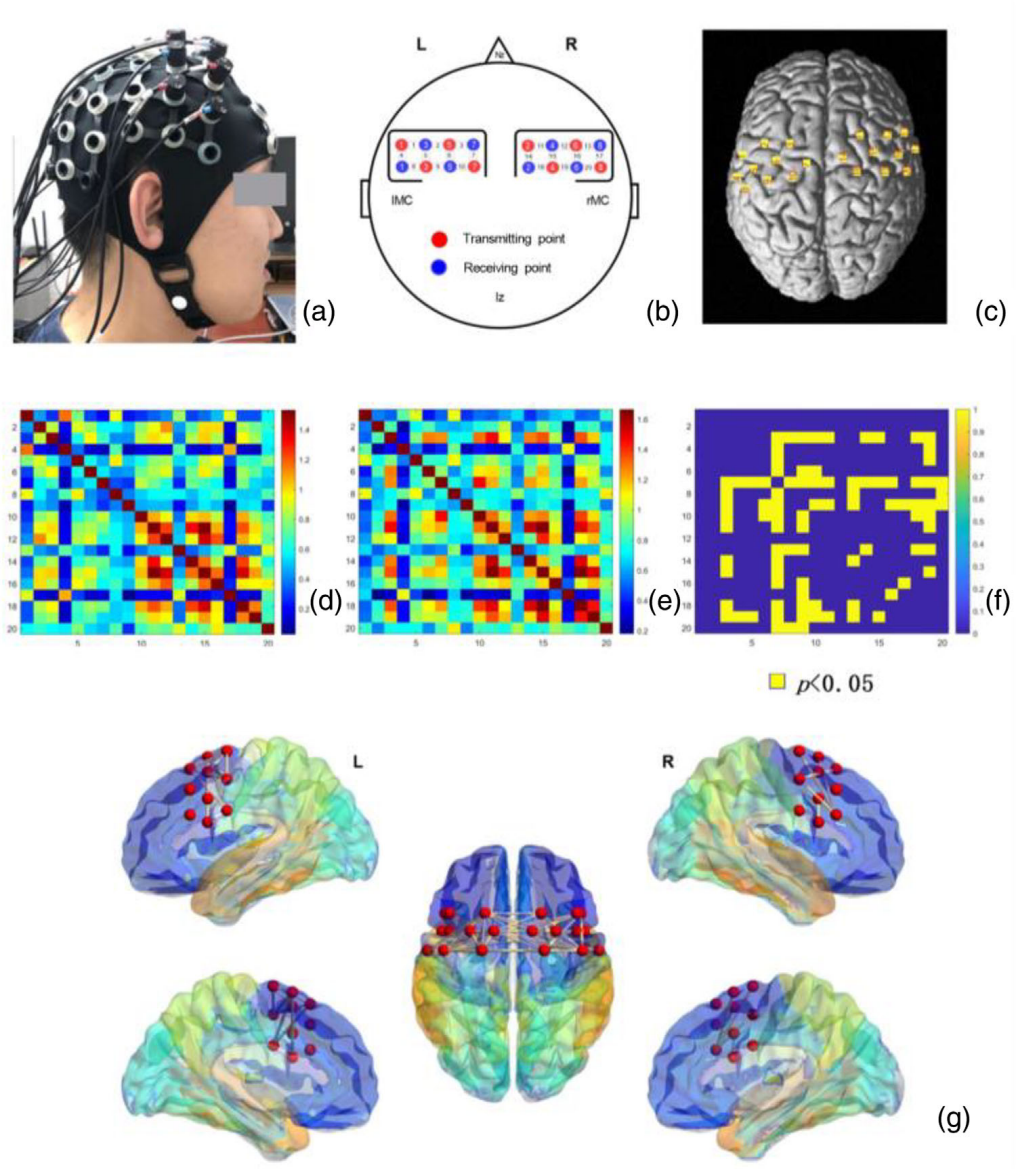
Resting state functional connectivity (RSFC) data presentation and imaging of brain regions (HbO signal). *Note*: (a) photopolar cap wearing. (b and c) Spatial distribution and alignment of near infrared spectroscopy (fNIRS) measurement channels. 4 × 2 (R, right), 4 × 2 (L, left) probe set configuration, 8 detectors (blue), 8 light sources (red), and 20 channels (white). lMC for left motor cortex; rMC for right motor cortex, Nz for nasal root; Cz for vertex, lz for posterior occipital rudiment. (d and e) RSFC matrices for two groups. (f) Matrix of *p* values for each “edge” in the two groups (HbO signal, *p* < .05, FDR‐corrected). (g) Distribution of brain regions of edges with intergroup differences (*p* < .05).

Comparisons were made between the groups for single edges of motor cortex (*n* = 190). The results revealed that: (1) at HbO signal level, there were 50 edges with significant differences (*p* < .05, 14 after FDR correction), including 15 edges in the right motor cortex, 8 on left, and 27 remotely connected between left and right, see Figure 4g, (2) at HbR signal level, there were 35 edges with significant differences (*p* < .05, 0 after FDR correction), including 14 edges on the right, 3 on left, and 18 remotely connected on left and right, (3) at HbT signal level, total of 110 edges differed significantly (*p* < .05, 73 after FDR correction), including 28 edges on the right, 22 on the left, and 60 remotely connected on left and right. At all three signal levels, the highest total number of RSFC difference “edges” was between left and right motor cortex, and total number of difference “edges” in left motor cortex was higher than in the right for both groups, see Table 3.

**TABLE 3:** Distribution of channels with intergroup differences of resting state functional connectivity (RSFC) in motor cortex (FDR corrected).

	Left motor cortex	Right motor cortex	Between the left and right motor cortex
Blood oxygen signals	Channels	Channels	Channels
HbO signal	CH7–CH3, CH10–CH3, CH8–CH7, CH9–CH7, CH10–CH7, CH10–CH9	–	CH11–CH3, CH14–CH3, CH18–CH3, CH11–CH7, CH14–CH7, CH15–CH7, CH11–CH7, CH18–CH7, CH19–CH7
Subtotal	6	0	8
HbR signal	–	–	–
Subtotal	0	0	0
HbT signal	CH2–CH1, CH3–CH1, CH6–CH1, CH7–CH1, CH8–CH1, CH9–CH1, CH2–CH1, CH10–CH1, CH5–CH 3, CH6–CH3, CH7–CH3, CH10–CH3, CH7–CH4, CH8–CH4, CH10–CH4, CH7–CH5, CH9–CH5, CH10–CH5, CH7–CH6, CH10–CH6, CH8–CH7, CH10–CH7, CH9–CH8, CH10–CH8	CH13–CH11, CH16–CH11, CH17–CH11, CH18–CH11, CH19–CH11, CH13–CH12, CH14–CH13, CH15–CH13, CH16–CH13, CH18–CH13, CH19–CH13, CH19–CH15, CH17–CH16, CH17–CH16 CH20–CH16	CH11–CH1, CH12–CH1, CH15–CH1, CH16–CH1, CH18–CH1, CH11–CH3, CH13–CH3, CH14–CH3, CH16–CH3, CH17–CH3, CH18–CH3, CH19–CH3, CH19–CH3, CH13–CH4, CH14–CH4, CH14–CH5, CH18–CH5, CH19–CH5, CH13–CH6, CH19–CH6, CH11–CH7, CH12–CH7, CH13–CH7, CH14–CH7, CH15–CH7, CH16–CH7, CH17–CH7, CH18–CH7, CH19–CH7, CH20–CH7, CH16–CH8, CH20–CH8, CH20–CH9, CH11–CH10, CH13–CH10, CH19–CH10, CH20–CH10
Subtotal	23	14	36

### 3.4 Test‐retest reliability assessment

Studies have revealed that RSFC based on fNIRS has better map‐wise and cluster‐wise intragroup consistency at group and individual levels (Zhang et al., 2011). In this study, subjects were asked to take the same test (retest) on third day (Box et al., 2020) after completing first test (test). The retest reliability was accomplished on RSFC values of high exercise level group and the control group (containing ICC mean values of three blood oxygen signal levels). Results exhibited that: (1) For total RSFC of motor cortex, *ICC* (*C*, 1) mean value was 0.40 ± 0.10, and *ICC* (*C*, *k*) mean value was 0.57 ± 0.11, indicating “general” reliability (0.40 ≤ ICC ≤ 0.58). (2) For RSFC of 190 “edges,” the *ICC* (*C*, 1) mean value was 0.88 ± 0.06, and *ICC* (*C*, *k*) mean value was 0.94 ± 0.03, both depicting “excellent” reliability (ICC ≥ 0.75), see Table 4.

**TABLE 4:** Cluster test‐retest reliability based on total resting state functional connectivity (RSFC) and 190 “edges.”

Blood oxygen signals	Group	Total RSFC cluster level			190 “edges” cluster levels	
		*ICC* (*C*, 1)	*ICC* (*C*, *k*)		*ICC* (*C*, 1)	*ICC* (*C*, *k*)
HbO	H Group	0.47	0.64		0.96	0.98
	L Group	0.41	0.59		0.88	0.94
HbR	H Group	0.42	0.59		0.85	0.92
	L Group	0.45	0.62		0.79	0.88
HbT	H Group	0.21	0.35		0.92	0.96
	L Group	0.44	0.61		0.90	0.95
Mean value	–	0.40 ± 0.10	0.57 ± 0.11		0.88 ± 0.06	0.94 ± 0.03

**FIGURE 5:**
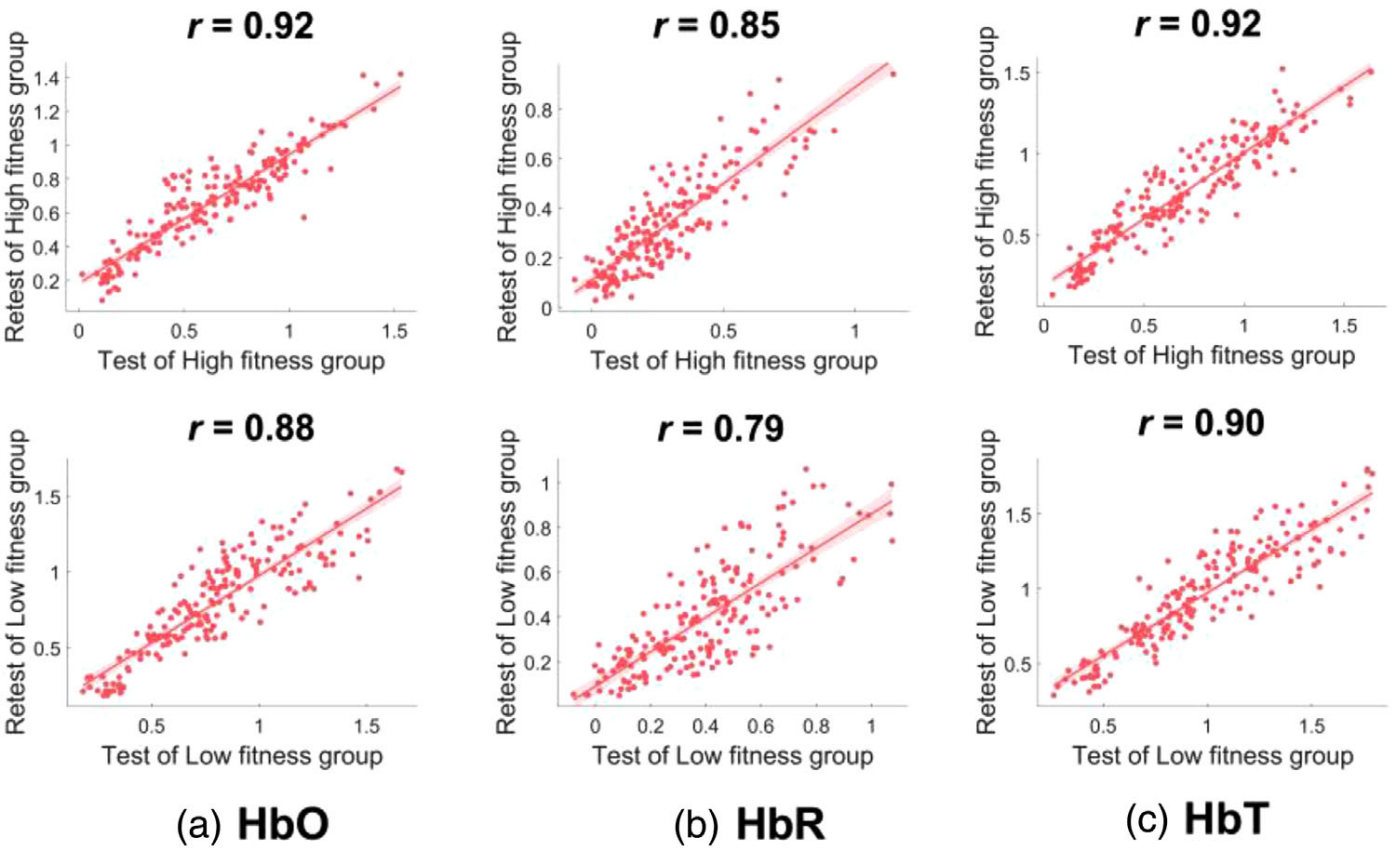
Results of test and retest correlation analysis for 190 edges of motor cortex. *Note*: Parts (a–c) explain scatter plots of 190 edges for H Group (top) and L Group (bottom) at HbO, HbR and HbT signal levels, respectively. Horizontal coordinates for resting state functional connectivity (RSFC) values of test, and vertical coordinates for RSFC values of retest; the solid diagonal line is fitted line, the light red area on either side of solid diagonal is CI confidence interval.

Correlation analysis of the test and retest RSFC data for 190 edges of motor cortex revealed that H Group (HbO: *r =* .92, *p = .000*, HbR: *r =* .85, *p = .000*, HbT: *r* = .92, *p =* .000) and L Group (HbO: *r* = .88, *p = .000*, HbR: *r* = .79, *p = .000;* HbT: *r* = .90, *p* = .000) had “excellent” reliability (*r* ≥ .75), see Figure 5.

## 4 DISCUSSION

This study was aimed to explore the differences of RSFC in motor cortex of the college students with high and low fitness levels. There was significant difference of total RSFC in motor cortex of the two groups. Total RSFC was lower in high fitness group compared to the low fitness group. Following issues were investigated regarding the data preprocessing and statistical analysis: (1) how to exclude noisy signals effect on RSFC? (2) whether adjacent cortical signals had impact on RSFC in motor cortex, and (3) whether results had good intragroup consistency (ICC).

First, the signal quality was ensured. In the data preprocessing stage, more stringent quality control was applied to the raw data, requiring SNR of no less than 5 (the system default value was 2) for each channel of participants. Data of substandard SNRs and high head movements were excluded. Second, the statistical tests were conducted in separate rows for avoiding the interference from other cortical signals. The anatomical locations covered by six channels in the first row of study included premotor cortex, supplementary motor cortex, dorsolateral prefrontal cortex, and insula capitata, see Table 2 and Figure 2. Results displayed no intergroup differences in mean RSFC values of 15 edges comprising the first row, which proposed that the signals from non‐motor cortices covered by photopoles (dorsolateral prefrontal cortex, insula capitata) had an impact on the RSFC of first row. RSFCs in second (supplementary motor cortex and premotor cortex) and third rows (primary sensorimotor cortex, primary motor cortex, premotor cortex, and supplementary motor cortex) (see Figure 2) were consistent with the total RSFC. Overall, the signal interference from other brain regions included in the study, with total mean of RSFC in motor cortex, is in acceptable limits.

The ICC exhibited that the RSFC had good retest reliability. Consistent with the Zhang et al. (2011) results, ICC in this study had “fair to good” range. It was found (Zhang et al., 2011) that RSFC in sensorimotor cortex had high remeasurement reliability at individual and cluster levels and within and between groups. ICC and correlation analysis results (mean values of three blood oxygen signals) were similar for 190 edges in motor cortex (H Group: ICC (*C*, 1) = 0.91, ICC (*C*, *k*) = 0.95, *r* = .90; L Group: ICC (*C*, 1) = 0.86, ICC (*C*, *k*) = 0.92, *r* = .86). These outcomes were in accordance with the previous study (Niu et al., 2013). This implies that fNIRS‐based RSFC is a valid biomarker for evaluating the fitness level.

In brain network studies, brain imaging techniques, such as functional magnetic resonance (fMRI) or electroencephalography (EEG), were employed for examining the relation of RSFC with fitness level. Raichlen et al. (2016) through fMRI study found that compared to healthy control group, the functional connectivity between frontoparietal network and brain areas, indirectly related the cognitive functions in frontal lobes (e.g., working memory) of athletes group. There was negative correlation of default mode network with brain regions linked to motor control (paracentral area), somatosensory function, and visual association ability in athletes. They also found that RSFC was lower between the motor network seed point and areas near posterior cingulate cortex in athletes’ group (Raichlen et al., 2016). Boyne et al (2018) applied fMRI for verifying the link between RSFC changes in brain regions and walking ability. The walking ability was positively correlated with stronger RSFC between the superior frontal gyrus and midbrain motor areas adjacent to anterior cingulate cortex but negatively correlated with weaker RSFC between cerebellar motor areas and motor cortex (Mary et al., 2016). A study conducted by Mary et al. (Seidler et al., 2015) noted that RSFC in striatum and striatal neuronal networks was correlated with the performance levels of implicit motor sequence learning. Study also depicted that the RSFC values in motor cortex of children and adults assisted in predicting the individual motor ability. Wu et al. (2014) utilized high density array EEG for measuring the RSFC among premotor cortex, primary motor cortex, and parietal cortex to examine the differences in learning task of visuomotor tracking. In general, fitness level (e.g., skill level, skill learning ability) was correlated with RSFC of local brain areas. There might be differences in RSFC of various fitness levels. So, why would the motor level cause differences in RSFC of brain regions?

Physical activity improves cognitive functions, such as attention (Bavelier et al., 2012; Patriat et al., 2013), logical reasoning (Bergman et al., 2011; Mackey et al., 2013), and working memory (Klingberg, 2010; Morrison et al., 2011). This improvement involves complex set of neural mechanisms like the specific changes in RSFC of human brain as induced by physical activity. RSFC changes occur because of the repetitive involvement of neural circuits in higher cognitive activities over time (Mackey et al., 2013; Takeuchi et al., 2013; Takeuchi et al., 2014) and that the “historical traces” of co‐activation between brain regions (every brain region leaves “traces” of co‐activation) are the basis of RSFC changes (Buckner et al., 2013; Keller et al., 2016). RSFC differences between populations can be due to the concentrated expression of “historical traces” among brain regions after prolonged engagement in a cognitive task (Duan et al., 2012). The intergroup differences in RSFC can be because of the changes in short range functional connectivity of motor cortex, and the remote functional connectivity between motor cortex and other brain regions related to motor cognitive demands generated due to the chronic physical activity in high fitness group (Raichlen et al., 2016). Song et al. (2020) analyzed RSFC characteristics of visuomotor areas in chess experts and found that they had decreased functional connections between right dorsal‐anterior subregion and left angular gyrus and increased between the right ventral‐anterior visual motion subregion and right superior temporal gyrus. Lu et al. (2018) compared the differences in spontaneous brain activity and functional connectivity of sensorimotor system for social dancers and nondancers. The dancers had decreased RSFC in the seed points of inferior frontal gyrus with bilateral insula, right inferior temporal gyrus, bilateral precentral gyrus, left postcentral gyrus, left middle temporal gyrus, left syrinx, and right cerebellum. Lu et al. (2018) suggested that the specific training experiences associated with ballroom dancing, including the high volume movement perception, attentional control, and motor regulation reduced the functional connections strength between brain regions, and thus conserved the neural resources in resting state. The lower RSFC in college students having high fitness level may be caused due to the sparing of neural resources at rest. Brain neurons in resting state wait for the task requests and this state is organized in a specific functional network. Brain activates the cooperation mechanism when a specific cognitive task is initiated to reallocate the limited resources of each brain region (Alonso et al., 2014).

“Low RSFC” in motor cortex of high fitness group is like the phenomenon of “athlete's heart” described in physiology. Athlete's heart theory states that the athlete's heart rate is lower at quiet; however, heart rate reserve is enhanced, which improves the heart's capability for adapting to higher exercise loads. Similarly, participants of high fitness level have lower RSFC but may have greater “RSFC reserve” for the brain flexibility to turn on the cognitive activity for meeting the cognitive demands matched by motor skills of varying complexity. These results are consistent with the neural efficiency model (Rypma et al., 2002). Compared to low fitness group, high fitness group has higher motor skill automation capability. The motor cortex uses lesser neural resources for completing the cognitive process, such as planning, execution, and control (Postle, 2015). It is thus an important direction to evaluate FC changes in people of diverse exercise levels during cognitive or motor tasks.

The results of 190 “edges” revealed that the number of edges having intergroup differences of local motor areas was different. At HbO and HbT signal levels, the number of edges with intergroup differences between left and right motor cortex were the most, followed by left motor cortex and right motor cortex, see Table 3 and Figure 4g. RSFC was important to understand the connectivity of large cortical networks (Lewis et al., 2009). If more brain regions were included in RSFC exploration, it was useful to recognize how physical activity could induce cortical communication from a distance and further explained how physical activity improved the motor skills and related cognitive abilities (Leopold et al., 2003). The interhemispheric asymmetries in motor cortex were linked with handedness (Amunts et al., 2000). All participants in this study were right‐handed. RSFC differences in left motor cortex can be greater, whereas RSFC in right motor cortex was more similar (Medvedev, 2014). Therefore, number of “edges” differing between the groups in left motor cortex was greater than in right motor cortex.

Examining the motor cortex only is the limitation of this study. The locomotion control is dependent on multitudinous brain regions and networks beyond the motor cortex, such as brainstem, spinal cord, cerebellum, basal ganglia, and frontal lobe cortex. A limited portion of cortical areas could be recorded because of the fNIRS limitations. So, RSFC differences in motor cortex observed through this study can be limited. Moreover, it is likely that the changes in brain structure and functions may differ among sports (Nakata et al., 2010). The characteristic RSFC performance in cerebral cortex of athletes engaged in various exercise types is worthy of further study. Scientific methods of determining fitness levels are lacking and only the athletic skill level certificate is used for grouping. More physiological indicators (i.e., in VO2 peak values) can be introduced as grouping criteria in future. It may be important to evaluate FC changes in people of diverse exercise levels during cognitive or motor tasks, which could assess the results of resting state connectivity. Furthermore, the effects of exercise or cognitive load on RSFC in motor cortex or other brain regions can be examined, such as the effect of acute exercise, short‐term exercise interventions, and working memory task on RSFC. Finally, the small sample pool and heterogeneity of participant characteristics limit the generalization and interpretation of these results for the healthy adults. This is true for the secondary analyses performed on the role of fitness level in healthy adults. A broader sample pool of fitness levels is imperative in validating the preliminary findings of this study.

## 5 CONCLUSION

This study aims at exploring the potential role of fitness level in RSFC. The evidence indicates that fitness levels cause specific changes in RSFC of motor cortex, which is a valid biomarker in evaluating the movement levels.

### PEER REVIEW

The peer review history for this article is available at https://publons.com/publon/10.1002/brb3.3099.

## Data Availability

The data that support the findings of this study are available from the corresponding author upon reasonable request.
